# Numbing the Pain: How Alcohol Use for Chronic Pain Relief Leads to Dependency – Review and Case Study

**DOI:** 10.1192/j.eurpsy.2025.947

**Published:** 2025-08-26

**Authors:** Â. Ferreira, I. Pereira, A. Falcão, J. Teixeira

**Affiliations:** 1 São Bernardo Hospital, Setúbal; 2 Júlio de Matos Hospital, Lisbon, Portugal

## Abstract

**Introduction:**

Chronic pain is a debilitating condition affecting 20% to 30% of adults globally, with prevalence rates rising to 19% to 38% in Europe. It is often linked to self-medication, particularly through alcohol consumption, due to alcohol’s short-term analgesic properties, which act on the opioid system. However, continued alcohol use for pain relief can lead to alcohol use disorder (AUD), worsening the pain and causing physical and mental health issues.

**Objectives:**

This study explores the relationship between alcohol use in chronic pain management and the development of AUD through a literature review and a clinical case.

**Methods:**

A narrative literature review was conducted using PubMed with the terms “alcohol use disorder” and “chronic pain.” English and Portuguese articles from the last 10 years were included, yielding 85 results. A clinical case involving a patient with chronic pain and alcohol dependence illustrates the issue.

**Results:**

The review shows that 28% to 35% of chronic pain patients use alcohol for symptomatic relief. While alcohol may provide initial pain relief, prolonged use leads to tolerance, increased consumption, and a significant risk of AUD. Approximately 18% to 25% of patients meet the criteria for AUD, with men being more commonly affected.

The clinical case describes a 56-year-old man with family history of AUD, who developed chronic pain after a work accident resulting in multiple trauma. The patient began consuming alcohol daily for pain relief, which escalated to an AUD diagnosis and worsened pain due to alcohol-induced hyperalgesia.

**Conclusions:**

Alcohol use is prevalent among chronic pain patients, often as a form of self-medication. However, this practice is frequently counterproductive, as increased consumption to counteract tolerance can lead to serious complications, including hyperalgesia, psychiatric disorders, and significant difficulties in managing both pain and alcohol use disorder (AUD). The clinical case highlights these issues and reinforces the need for a multidisciplinary approach that addresses both pain management and alcohol dependence.

Early detection of problematic alcohol use is essential to prevent the development of AUD. A comprehensive treatment plan, incorporating strategies for pain control and addiction management, is crucial for improving the overall health and well-being of patients dealing with chronic pain.

**Disclosure of Interest:**

None Declared

